# Real world drug treatment models for pregnancy complicated with urinary tract infection in China from 2018 to 2022: a cross-section analysis

**DOI:** 10.3389/fphar.2024.1349121

**Published:** 2024-01-29

**Authors:** Jing Jin, Changyan Li, Yuqing He, Jiaqian Pan, JiaLei Zhu, Jing Tang

**Affiliations:** Department of Pharmacy, The Obstetrics and Gynecology Hospital of Fudan University, Shanghai, China

**Keywords:** China, cross-sectional analysis, urinary tract infections during pregnancy, drug treatment models, real world

## Abstract

**Objective:** Urinary tract infection (UTI) is common in pregnant women. The selection of anti-infection plans during pregnancy must take into account the dual factors of patient pregnancy status and urinary tract infection anti-infection treatment, as well as the efficacy, cost, risk, and potential adverse reactions associated with each method applied to individual patients. Consequently, there are numerous drugs from which to choose; presently, there is no unified conclusion regarding the choice of drug therapy, and there is a lack of long-term drug treatment for UTI during pregnancy. Our objective is to investigate the actual drug treatment patterns of UTI patients during pregnancy in China over the past 5 years, with a particular emphasis on the trend and rationality of antibiotic use in these patients over the past 5 years.

**Method:** We conducted a cross-sectional analysis of data from a China Medical Association-supervised hospital prescription analysis cooperation initiative. From January 2018 to December 2022, the information is extracted from prescriptions/medical orders of patients with UTI during pregnancy. Using a primary anatomical therapeutic chemistry (ATC) classification code and the US Food and Drug Administration (FDA) classification, we quantified the frequency of drug use and drug types. We also calculated the prevalence of the most frequently prescribed antibacterial medications and assessed the efficacy of anti-infection plans based on drug labels and guidelines.

**Results:** Among the 563 patients included in this research, Chengdu (36.59%), Guangzhou (27.72%), and Shanghai (8.70%) were the top three cities. Over the course of 5 years, the average age was 29.60% ± 6.59 years, with approximately 60.21% of women between the ages of 25 and 34. Each patient’s primary anti-infection medications were statistically analyzed. Cephalosporins (403, 71.58%), enzyme inhibitors (66, 11.72%), and penicillins (34, 6.04%) were the first few categories, followed by the most commonly used cephalosporins. Cefuroxime, ceftriaxone, and cefdinib, rounded out the top five. Cefoxitin and cefaclor. According to the 5-year change in dosage, cephalosporins have always ranked first. Three of the top five most expensive drugs are cephalosporins, carbapenems, and enzyme inhibitors. Teicoplanin, tigecycline, nifurtel, linezolid, and quinolones ranked among the top five in terms of per-patient drug costs for patients receiving comprehensive treatment drugs.

**Conclusion:** In the 5 years of research, the average age of patients who visit a doctor has not increased substantially, but the opportunity cost of female fertility has increased, which has severely impeded the fulfillment of fertility desires. The selection of medications is generally reasonable, and the dosage of the first-line cephalosporins recommended by the guidelines is relatively high in this study. The dosage of furantoin and fosfomycin, which are more prevalent in urinary tract infections, is however relatively low. In addition, some expensive pharmaceuticals may increase patients’ financial burden. On the premise of meeting clinical needs, future research will focus on how to further improve the level of rational drug use in outpatient clinics, attain economical, safe, and effective drug use, and thus reduce the economic burden on patients.

## 1 Background

Urinary tract infections (UTIs) are common in expectant women, and even asymptomatic infections can cause severe complications for the mother and fetus, including low birth weight, premature birth, stillbirth, preeclampsia, maternal anemia, sepsis, and amnionitis (Bigna et al., 2018; Tchatchouang et al., 2019; Ali et al., 2022). Increased likelihood of developing a UTI during pregnancy is attributable to alterations in expectant women’s physiology and lowered immunity (Delzell and LeFevre, 2000; Hill et al., 2005; Haider et al., 2010; Kalinderi et al., 2018; Storme et al., 2019; Getaneh et al., 2021).

Current research is more concerned with the etiology, bacterial spectrum, and drug sensitivity of urinary tract infections in expectant women (Belete, 2020; Belete and Saravanan, 2020; Getaneh et al., 2021; Ali et al., 2022). The guidelines for drug treatment regimens and follow-up after mode treatment for UTI in pregnancy are consistent, but there are still inconsistencies, such as prenatal screening for bacteriuria and the use of fluoroquinolones in lower or upper urinary tract infections (Corrales et al., 2022). A study of 1,140 pregnant women with ASB revealed that it is impossible to determine which drug is the most effective or safe for treating UTIs during pregnancy (Guinto et al., 2010). One study demonstrated the efficacy of long-term treatment with furantoin, while another suggested that ampicillin is better tolerated. There is no evidence to suggest the benefits or drawbacks of various dosing regimens, which should be thoroughly considered. This gives us ideas for future investigation. There are still substantial disparities in the specific drug selection practices of various countries around the world, particularly in China, where there is no current consensus and authoritative literature reports. The Consensus of Chinese Women’s Urinary Tract Infection Diagnosis and Treatment Experts points out that there is no unified opinion on the selection and treatment course of antibiotics for gestational urinary tract infections. Drugs should be selected based on urine bacterial culture and sensitivity, while considering the safety and effectiveness of medication for both the mother and fetus. The recommended drugs mainly include penicillin, cephalosporins, etc (China Medical Women’s Association, 2017). In clinical practice, obstetricians must increasingly consider how to use anti-infective medications rationally and safely with expectant patients. By analyzing actual data from 2018 to 2022, we hope to close this knowledge gap. Our objective is to investigate the actual drug treatment patterns of UTI patients during pregnancy in China over the past 5 years, with a particular emphasis on the trend and rationality of antibiotic use in these patients over the past 5 years.

## 2 Methods

The data comes from the China Medical Association’s Hospital Prescription Analysis Cooperation Project, which collects prescription/order data from nearly 120 hospitals in Beijing, Chengdu, Guangzhou, Harbin, Hangzhou, Shanghai, Shenyang, Tianjin, and Zhengzhou from 2018 to 2022 on a quarterly basis. This project provides the following data: time, city, hospital code, medication route, dosage, unit cost, medication frequency, single dose, quantity, age, and initial diagnosis.

This study collected outpatient and inpatient prescription/medical advice data for “pregnancy”, “pregnancy”, and “urinary tract infection” from 1 January 2018 to 31 December 2022. Other diagnoses, such as “ectopic pregnancy”, “adverse pregnancy history”, “infertility”, and patients with urinary tract infections unrelated to pregnancy were excluded. This project only counts western antibiotics; traditional Chinese patent medicines and simple preparations, herbal medicine, and other drugs extraneous to urinary tract infection treatment are excluded.

For further analysis, we divided patients into various age groups and geographic regions, screened the most important treatment medications, and conducted additional analysis based on drug selection, administration route, drug dosage, *etc.* According to the pharmacological classification of therapeutic drugs, calculate the sales revenue of drug consumption in the past 5 years and calculate the proportion of sales revenue to total sales. Concurrently, we adhere to the World Health Organization (WHO) and Defined Daily Dose (DDD) system. The DDD value is determined using the “Clinical Medication Guidelines” (2010 edition) and “New Pharmacology” (17th edition) (National Pharmacopoeia Commission, 2010), in conjunction with the clinical medication situation and drug instructions. To conduct a rationality analysis, we evaluated the complete frequency and single dose information of hormone prescriptions in accordance with the recommended drug labeling protocol and the most recent Chinese guidelines.

Statistical analysis was conducted using Excel 2013 and SPSS software (version 25; SPSSInc., Chicago, IL, United States). Continuous variables are shown as mean ± standard deviation. Categorical variables are presented as numbers and percentages. Demographic and prescription information was grouped into counts.

## 3 Results

### 3.1 Patients’ demographic characteristics

**FIGURE 1:**
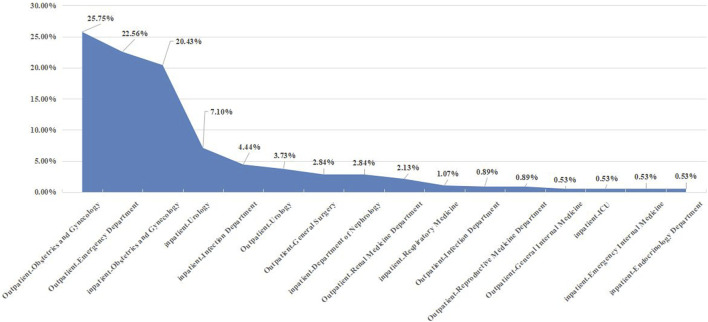
Proportion of outpatient/inpatient departments.

We included 342 outpatient and 221 inpatient patients among 563 patients in this experiment based on the inclusion criteria, including prescription data such as region, reimbursement method, and expenses. Chengdu (36.59%), Guangzhou (27.72%), and Shanghai (8.70%) are the top three cities. Over a 5-year period, the average age was 29.60 6.59 years, with roughly 60.21% of women aged 25–34 years. Table 1 shows the demographic features of the patients. The main outpatient departments are Obstetrics and Gynecology (25.75%), Emergency (22.56%), and Urology (3.73%), whereas the main inpatient departments are Obstetrics and Gynecology (20.43%), Urology (7.10%), and Infection (4.44%). Figure 1 shows the departments that have three or more patients.

**TABLE 1:** Demographic characteristics of the patients (*n* = 563).

Year	2018	2019	2020	2021	2022	Total
Region, n (%)						
Beijing	6 (1.07%)	8 (1.42%)	8 (1.42%)	10 (1.78%)	3 (0.53%)	35 (6.22%)
Chengdu	33 (5.86%)	58 (10.30%)	36 (6.39%)	37 (6.57%)	42 (7.46%)	206 (36.59%)
Guangzhou	30 (5.33%)	40 (7.10%)	25 (4.44%)	34 (6.04%)	27 (4.80%)	156 (27.72%)
Haerbin	3 (0.53%)	3 (0.53%)	0 (0.00%)	5 (0.89%)	4 (0.71%)	15 (2.66%)
Hangzhou	4 (0.71%)	4 (0.71%)	9 (1.60%)	7 (1.24%)	13 (2.31%)	37 (6.57%)
Shanghai	7 (1.24%)	9 (1.60%)	9 (1.60%)	15 (2.66%)	9 (1.60%)	49 (8.70%)
Shenyang	0 (0.00%)	0 (0.00%)	0 (0.00%)	5 (0.89%)	0 (0.00%)	5 (0.89%)
Tianjin	5 (0.89%)	4 (0.71%)	5 (0.89%)	2 (0.36%)	0 (0.00%)	16 (2.84%)
Zhengzhou	6 (1.07%)	4 (0.71%)	7 (1.24%)	9 (1.60%)	18 (3.20%)	44 (7.82%)
Tatol	94 (16.70%)	130 (23.09%)	99 (17.58%)	124 (22.02%)	116 (20.60%)	563 (100%)
Drug costs	13,342.24 (16.08%)	18,420.57 (22.20%)	11,125.53 (13.41%)	20,861.09 (25.14%)	19,234.43 (23.18%)	82,983.86 (100%)
Cost per patient	141.94	141.70	112.38	168.23	165.81	147.40
Age, n (%)						
18-24	19 (3.37%)	21 (3.73%)	14 (2.49%)	33 (5.86%)	22 (3.91%)	109 (19.36%)
25-34	61 (10.83%)	82 (14.56%)	63 (11.19%)	60 (10.66%)	73 (12.97%)	339 (60.21%)
35-44	14 (2.49%)	26 (4.62%)	21 (3.73%)	29 (5.15%)	17 (3.02%)	107 (19.01%)
45-50	0 (0.00%)	1 (0.18%)	1 (0.18%)	2 (0.36%)	4 (0.71%)	8 (1.42%)
Average	29.02 ± 4.74	29.67 ± 6.04	30.26 ± 5.91	29.10 ± 8.72	29.98 ± 6.40	29.60 ± 6.59

### 3.2 Drug classes used by patients

**FIGURE 2:**
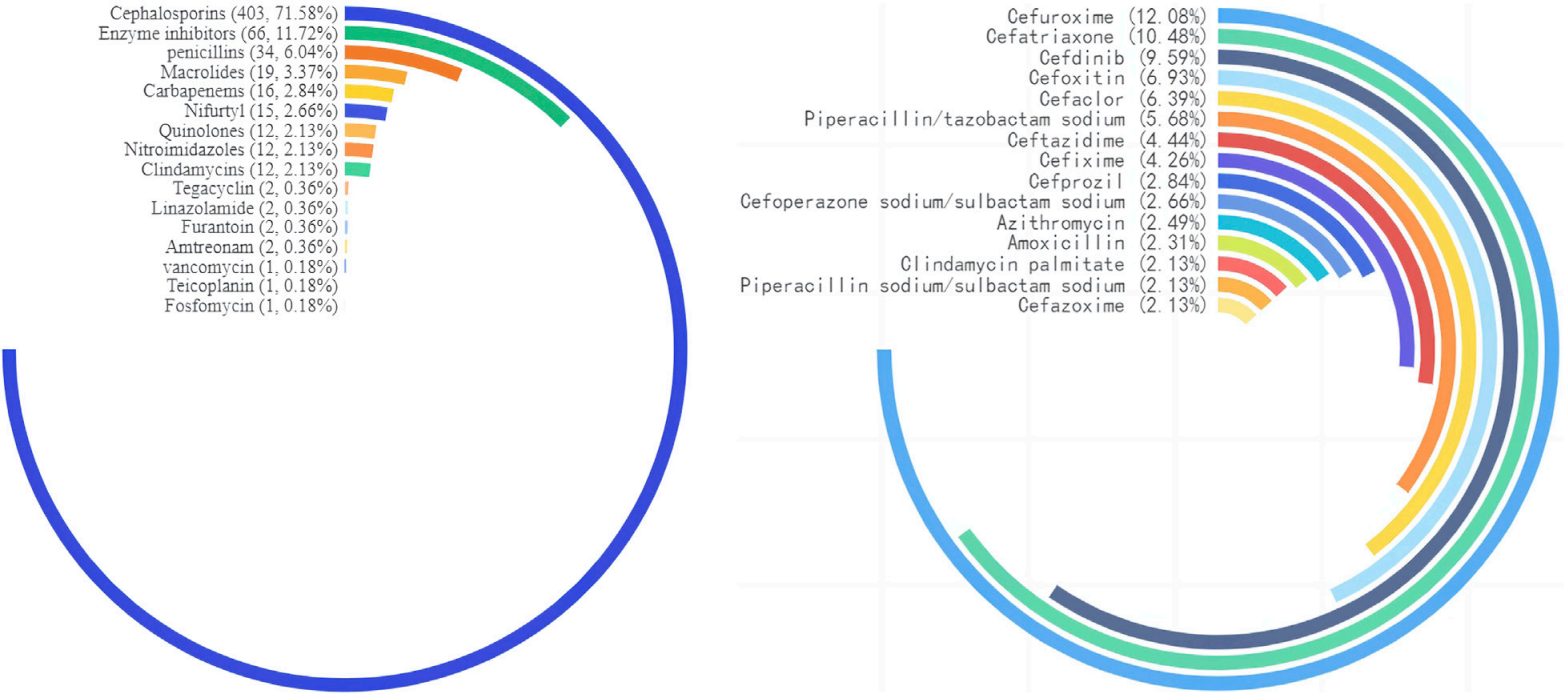
Circular bar chart map of drug type distribution.

**FIGURE 3:**
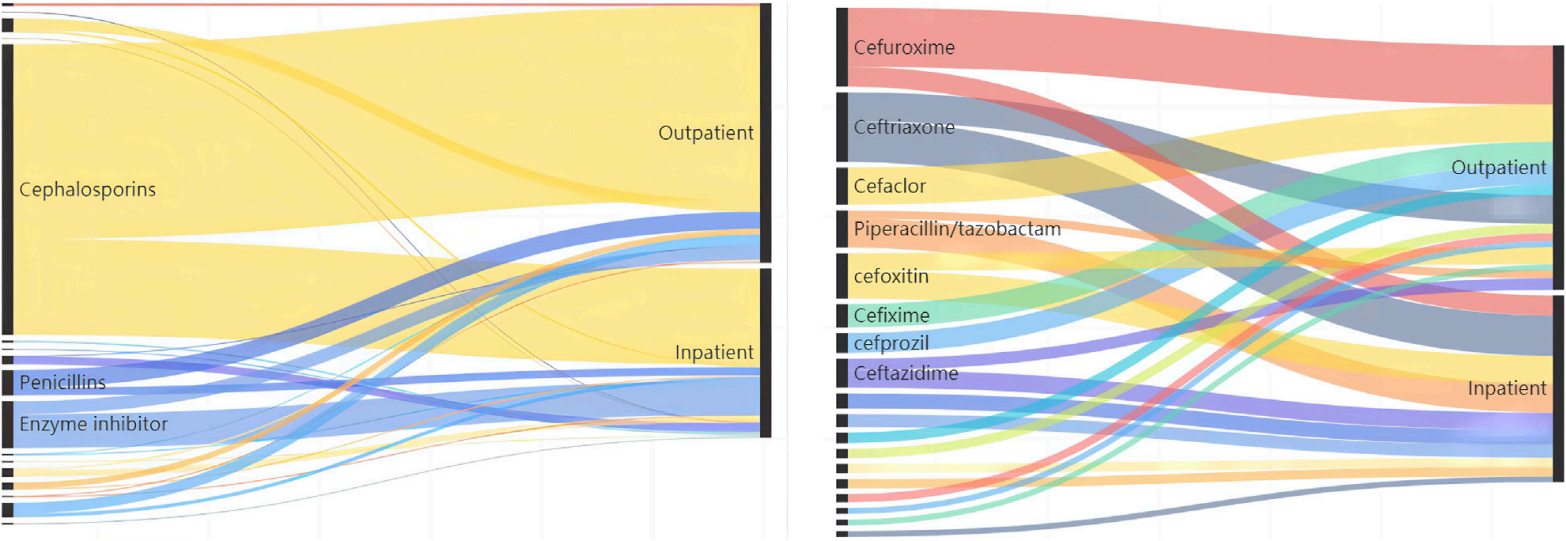
Top three anti-infective drug type/top ten anti-infective drug detected in the outpatient/inpatient patients.

**FIGURE 4:**
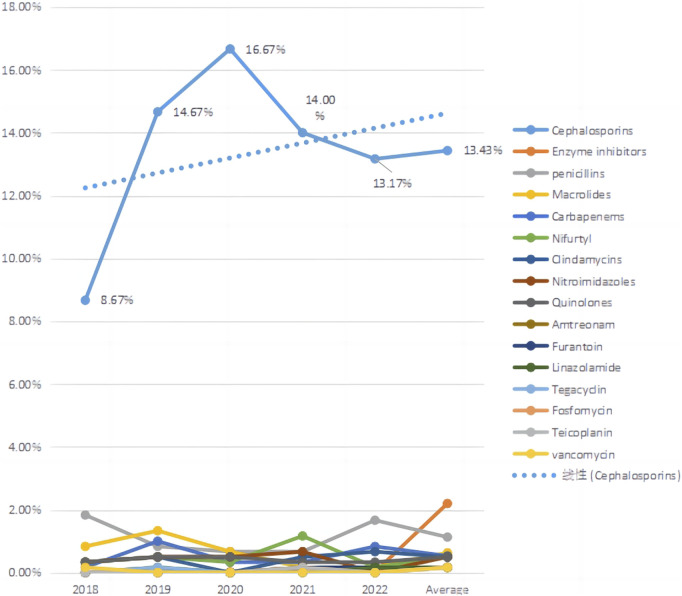
Trends in main therapeutic drugs use.

We counted the number of anti-infective medications used by each patient, and 37 of them used two types of antibiotics, including cephalosporins (403, 71.58%), enzyme inhibitors (66, 11.72%), and penicillin (34, 6.04%). The top five antibiotics were cefuroxime (68, 12.08%), ceftriaxone (59, 10.48%), and cefdinib (54, 9.59%), followed by cefoxitin (39, 6.93%) and cefaclor (36, 6.39%), as shown in Table 2. Figure 2 depicts the distribution of all categories, whereas Figure 3 depicts the matching outpatient/inpatient services. During the 5-year period, there was no substantial change in the type and dosage of antibacterial medications. Cephalosporins were the most commonly used antibiotics overall, as illustrated by the trend chart in Figure 4.

**TABLE 2:** Main therapeutic drugs of the patients (n = 563).

Drug classification	2018 (%)	2019 (%)	2020 (%)	2021 (%)	2022 (%)	Average (%)
Cephalosporins	8.67	14.67	16.67	14.00	13.17	13.43
Enzyme inhibitors	0.01	0.02	0.03	0.02	0.02	2.20
penicillins	1.83	0.83	0.67	0.67	1.67	1.13
Macrolides	0.83	1.33	0.67	0.17	0.17	0.63
Carbapenems	0.17	1.00	0.33	0.33	0.83	0.53
Nifurtyl	0.33	0.50	0.33	1.17	0.17	0.50
Clindamycins	0.33	0.50	0.00	0.50	0.67	0.50
Nitroimidazoles	0.33	0.50	0.50	0.67	0.00	0.50
Quinolones	0.33	0.50	0.50	0.33	0.33	0.50
Amtreonam	0.00	0.00	0.00	0.17	0.17	0.17
Furantoin	0.00	0.00	0.00	0.17	0.17	0.17
Linazolamide	0.00	0.17	0.00	0.00	0.17	0.17
Tegacyclin	0.00	0.17	0.00	0.00	0.00	0.17
Fosfomycin	0.00	0.00	0.00	0.17	0.00	0.17
Teicoplanin	0.00	0.00	0.00	0.17	0.00	0.17
vancomycin	0.17	0.00	0.00	0.00	0.00	0.17

### 3.3 Analysis of drug usage and dosage

**FIGURE 5:**
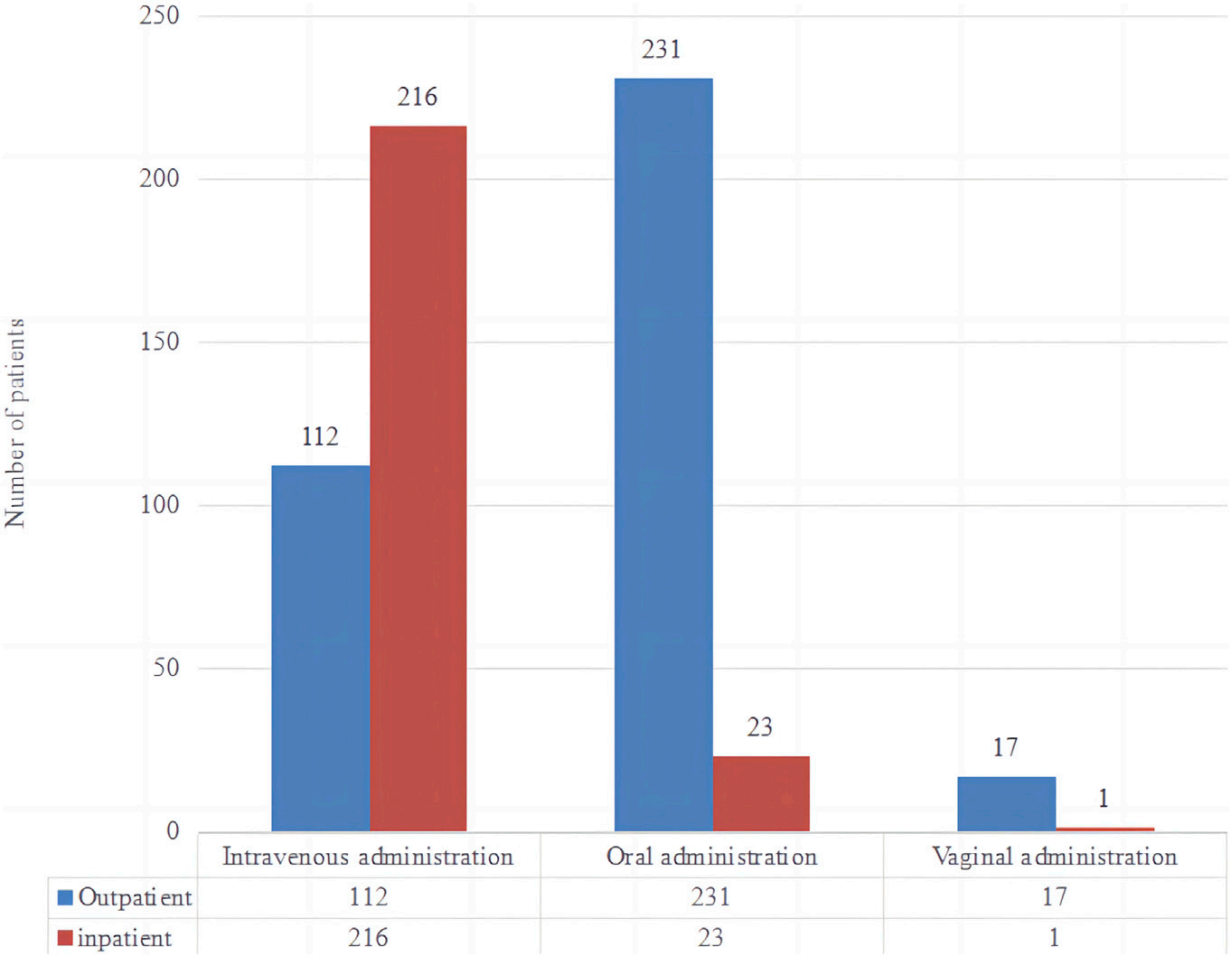
Amount of patient administration route.

**FIGURE 6:**
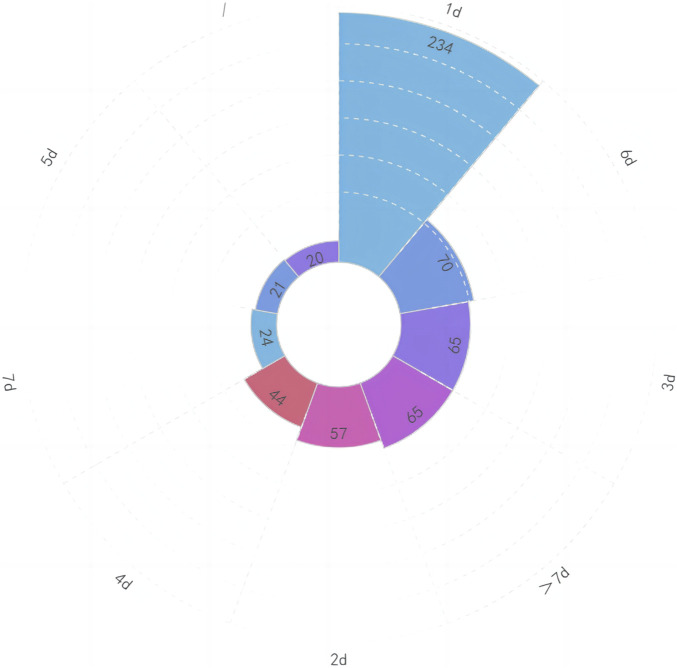
Amount of patient course of treatment.

When we analyzed the data, we discovered that there were primarily three types of medication based on the mode of administration: intravenous (328, 54.67%), oral (254, 42.33%), and vaginal (18, 3.00%). Please see Figure 5 for further information. According to the summary data, the dosage supplied was essentially reasonable. For a total of 20 patients, the combined usage and dosage were not given. The therapy period consists primarily of 1 day (234, 39.00%), 3 days (65, 10.83%), 5 days (21, 3.50%), and 7 days (24, 4.00%). Please see Figure 6 for further information.

### 3.4 Costs

We computed the total cost and cost *per capita* for patients utilizing various therapeutic medications. In terms of total cost, the top five medications were cephalosporins, carbapenems, enzyme inhibitors, quinolones, nifurtel, and the number of patients receiving comprehensive treatment drugs. The five most expensive medications *per capita* were cefuroxime, ceftriaxone, cefdinir, cefoxitin, and cefaclor. See Table 3 for details. Analyzing the data, it was found that the proportion of funds used for injection administration was 78.66%, the proportion for oral administration was 20.03%, and the proportion for vaginal administration was 1.32%.

**TABLE 3:** Cost of therapeutic drugs use per person each year from 2018 to 2022.

Main therapeutic drugs	2018	2019	2020	2021	2022	Amount	Frequency	Per capita
						(RMB)		(RMB)
Cephalosporins	6,548.17	9,136.52	8,170.11	8,881.42	4,813.26	37,549.48	403	93.17
Enzyme inhibitors	4,698.28	1,090.82	1829.78	4,183.2	0	11,802.08	66	178.82
penicillins	609.24	146.04	27.96	8.91	434.65	1,226.8	34	36.08
Macrolides	350.58	304.6	338.73	92.4	0	1,086.31	19	57.17
Quinolones	343.83	405.07	94.46	223	5,902.96	6,969.32	12	580.78
vancomycin	312.24	0	0	0	0	312.24	1	312.24
Carbapenems	259.2	4,033.14	311.98	503.68	7,527.48	12,635.48	16	789.72
Nifurtyl	164	174.86	131.72	2,346.51	41.4	2,858.49	15	190.57
Clindamycins	38.34	83.82	0	425.62	429.12	976.9	12	81.41
Nitroimidazoles	18.36	145.8	23.5	0	0	187.66	12	15.64
Tegacyclin	0	1800	0	0	0	1800	2	900.00
Linazolamide	0	1,420.56	0	0	0	1,420.56	2	710.28
Amtreonam	0	0	45.2	34.14	0	79.34	2	39.67
Furantoin	0	0	3.57	0.84	0	4.41	2	2.21
Teicoplanin	0	0	0	2015.55	0	2015.55	1	2015.55
Fosfomycin	0	0	0	48.56	0	48.56	1	48.56
Total	13,342.24	18,741.23	10,977.01	18,763.83	19,148.87	80,973.18	600	134.96

## 4 Discussion

### 4.1 Demography characteristics

In the past 5 years, the majority of patients were between the ages of 25 and 34, with an average age of 29.60 6.60 years, according to statistical data. The age of treatment did not increase significantly, and there was no statistically significant change in age (*p* > 0.01). 50%–60% of pregnancies are diagnosed with a urinary tract infection, making it one of the most prevalent infections during pregnancy (Baraka et al., 2021). It can be separated into lower urinary tract infections, such as asymptomatic bacteriuria (ASB) or acute cystitis (AC), and upper urinary tract infections, such as acute pyelonephritis (APN) (Glaser and Schaeffer, 2015). Estimates place the incidence of ASB in expectant women between 2% and 10% (Bonkat et al., 2018). The exported data on pregnancy combined with UTI is relatively small compared to literature reports, which may be attributable to different diagnostic writing habits in different hospitals or to the large number of ASB patients who did not seek medical attention in a timely manner; simultaneously, there are significant differences in the proportion of patients in different regions due to a variety of factors. This may be the result of various diagnostic criteria, inconsistent calculation criteria, or regional variations in prevalence rates. This study will not draw any premature conclusions.

The age of patients seeking medical treatment has not risen in the last 5 years, showing that patients’ fertility demands are primarily centered around the age of 30. To some extent, the adjustment of childbearing time conventions indicates that as society develops, the number of people suffering from environmental pollution, increased work pressure, and delayed marriage and childbirth does not gradually increase, and people’s ideological concepts are constantly improving and updating (Hu et al., 2004; Liang et al., 2021). Many women believe that age is a significant component in their childbirth decision-making and that they should complete the duty of delivering at a specific age. If they stray from this age convention and have difficulties giving birth, this becomes a major issue. The rising opportunity cost of women’s fertility has made it difficult for them to achieve their fertility goals.

### 4.2 Types of drugs

According to data on antibiotic use by patients in major cities around the country, we discovered that outpatient and inpatient patients have the greatest variety and proportion of cephalosporins. When selecting antibiotics during pregnancy, consider the mother’s and fetus’s safety. According to the US Food and Drug Administration (FDA), the majority of the antibacterial medications indicated in International Guidelines are Class B, which indicates no adverse responses have been observed in well-controlled human pregnancy trials. There are limited guidelines that specify treatment for ASB and cystitis (Bonkat et al., 2018; Kranz et al., 2017; Betschart et al., 2020; de Cueto et al., 2017; Caron et al., 2018; Institute of Obstetricians and Gynaecologists, 2018; NICE NG109, 2022; NICE NG111, 2022). This could be due to shifting patterns of worldwide antimicrobial resistance, which means that therapy should be based on urine culture and sensitivity recommendations in laboratory reports, while also taking into account the authorized use of antibiotics during pregnancy.

The guidelines propose using furantoin, fosfomycin, and amoxicillin as the first line of treatment, followed by cephalosporin and amoxicillin (NICE NG109, 2022; Martinez et al., 2014). Distinct countries have distinct antibiotic preferences. A study of doctors in Denmark, Finland, Norway, and Sweden found that β-Lactam antibiotics (particularly pimecillin) and nitrofurantoin are their first-line treatments. In the United States, amoxicillin is commonly used, although trimethoprim and furantoin are favoured in Canada. Penicillin and cephalosporins are recommended in the United Kingdom (Christensen, 2000). This study’s statistical material differs from earlier research in some ways. According to our findings, cephalosporins, penicillin, and enzyme inhibitors continue to have a significant advantage in the anti-infection therapy of pregnant women with UTI in China. This could be because cephalosporins have good safety data (China Medical Women’s Association, 2017), However, drugs such as furantoin, fosfomycin, and others with higher recommended usage levels in other countries are relatively lower in China (Gupta et al., 2011). Doctors may prescribe enzyme inhibitors to individuals who are suffering from severe symptoms.

The selection of medications for the treatment of UTIs during pregnancy must take the safety of the mother and fetus into account (Bookstaver PB et al., 2015). The majority of antibiotics can cross the placenta, and it is crucial to determine whether they will have negative effects on the fetus. However, there is limited research on the effects of medications on the fetus during pregnancy. The majority of information on drug safety comes from animal studies and observational studies. The FDA’s classification of pregnancy lacks high-quality data. Despite the dearth of evidence, numerous antibacterial drugs, such as penicillin, cephalosporin, clindamycin, etc., have been used for several years during pregnancy without adverse maternal or fetal effects. Meta-analysis did not prove which antibacterial drug is best for ASB and symptomatic UTIs; therefore, empirical treatment is determined based on antibacterial spectrum, antibacterial activity, and pathogen culture results, as well as the cost (Gupta et al., 2011; Widmer et al., 2015). Few drugs have been definitively demonstrated safe. Therefore, care should be taken to minimize the number of drugs used, only when the benefits outweigh the risks, selecting drugs with the best safety profile, and employing the lowest effective dose and shortest treatment course.

Penicillin has been widely used and its safety has been confirmed in many studies (China Medical Women’s Association, 2017), including penicillin G, ampicillin, and amoxicillin. Although bacterial resistance is common, it is still the most frontline treatment drug. Cephalosporins are also a class of antibiotics with high safety, and third-generation cephalosporins are commonly used in empirical treatment. Furantoin is very effective in treating lower UTI, and its safety is controversial. The US neonatal defect prevention study suggests that furantoin is associated with congenital malformations such as eye deformities, atrial septal defects, and cleft lip and palate. Looking at it correctly, only 35% of patients can recall the name of the medication used. ACOG believes that it should be used reasonably in the early stages of pregnancy, and can be used as a first-line treatment plan in the middle and late stages of pregnancy. Between 1999 and 2009, a total of 105,492 pregnant women were included, and a total of 6,561 fetuses and newborns were diagnosed with congenital malformations. The incidence of malformations was 5.7% (76 of 1,329) in the exposed group and 6.2% (6,485 of 104,163) in the unexposed group, with no statistically significant differences. Exposure to furantoin in early pregnancy did not increase the incidence of fetal malformations (Goldberg et al., 2013). Phosphomycin is a broad-spectrum antibacterial drug that plays an increasingly significant role in lower urinary tract infections. Trimethoprim sulfamethoxazole is not recommended as a first-line solution. The US neonatal defect prevention study suggests that SMZ is more teratogenic than other drugs, but other studies do not recognize it. ACOG believes that if there are no other drugs available in early pregnancy, this product can be chosen. In addition to teratogenesis, hyperbilirubinemia and nuclear jaundice may occur in late pregnancy.

A systematic evaluation in the Cochrane Database analyzed the most effective treatment methods for symptomatic UTI (cystitis and pyelonephritis) during pregnancy by analyzing the cure rate, recurrence rate, incidence of premature birth, and necessity for antibiotic replacement. The results showed that all the antibiotics studied were effective and had few complications. There is not enough evidence to recommend a specific treatment plan. This study shows that China places more emphasis on effectiveness and safety in the treatment process, with less use of drugs such as furantoin and fosfomycin. Further consideration may be needed on issues such as bacterial resistance (Keating, 2013).

### 4.3 Usage and dosage

In this study, cephalosporins and penicillin are first-line medications for the treatment of UTI during pregnancy in both outpatient and inpatient patients. A total of 20 patients’ usage and dosage were not specified. Physiological changes in the mother can affect pharmacokinetics and decrease serum drug concentration, increase intravascular and extravascular fluid volume, increase renal blood flow velocity, GFR, and increase fetal drug distribution. In our study, however, we discovered that the dosage provided to pregnant patients stayed at adult levels with no significant modifications. More research is needed to understand whether the dosage of UTI should be increased during pregnancy or adjusted dependent on body weight.

There appears to be no difference in treatment outcomes between 3-day short courses and 7-day long courses, and short courses can reduce costs and side effects, have higher compliance, and reduce fetal drug exposure (Gupta et al., 2011). In our study, the majority of patients were treated for a single day. This may be because the majority of patients had ASB or lower UTI, which did not progress to upper UTI. It may also be due to patients’ concerns regarding the influence of excessive antibiotic use on pregnancy outcomes.

### 4.4 Drug amount

Cephalosporins, carbapenems and enzyme inhibitors account for the highest amount of money used by patients with anti infective drugs. Teicoplanin, tigecycline and carbapenems rank first in terms of *per capita* cost. New anti infective drugs account for the highest *per capita* cost. The drugs with the top sales amount are commonly used drugs, including cephalosporin antibiotics. With the promotion of national volume procurement, many cephalosporin antibiotics have been included in the volume variety, which is relatively cheap compared to other types of drugs, but the frequency of use is also high. The *per capita* medication amount is significantly out of sync with DDDs, because these types of drugs are new types of antibiotics and special grade antibiotics, with relatively high prices. There is a certain trend in drug use in research data from different years. The changes in amount in different years and drug consumption structure show consistency in drug use in hospitals in different regions. It can be seen that everyone has a general consensus on the choice of main treatment drugs. The statistical results show that patients who receive intravenous medication have the highest proportion of medication costs, as compared to those who take oral medication, patients who use intravenous medication have a more severe condition, a larger dosage, and a longer course of treatment, all of which can lead to an increase in drug costs.

## 5 Conclusion

Our study counted the prescriptions/medical orders of patients with pregnancy complicated with UTI from 2018 to 2022. The average age of patients to see a doctor did not increase significantly from the 5 years of statistics. The opportunity cost of female fertility increased, which seriously hindered the realization of fertility desire. The overall medication selection is relatively reasonable, and the first-line cephalosporin antibiotics recommended by the guidelines are also used in relatively high amounts in this study. However, it should be noted that drugs recommended or used in other countries such as fosfomycin and furantoin are extremely low in China. However, our statistical data shows that some expensive drugs can increase the economic burden on patients. On the premise of meeting clinical needs, how to further improve the level of rational use of drugs in outpatient clinics, achieve economic, safe and effective use of drugs, and thus reduce the economic burden on patients will be the focus of future work.

For the treatment of UTIs during pregnancy, it is not possible to draw the conclusion of which drug is the most effective or safe. Empirical treatment is based on antibacterial spectrum, antibacterial activity, pathogenic results, and cost. In addition to well-established penicillin and cephalosporins, there is increasing evidence that furantoin, fosfomycin, and sulfonamide drugs can be applied to UTIs.

The follow-up after ASB treatment includes close supervision and prophylactic treatment with antibiotics, and there is currently no evidence to recommend which regimen is the optimal for preventing recurrence of UTI during pregnancy.

## Data Availability

The original contributions presented in the study are included in the article/Supplementary Material, further inquiries can be directed to the corresponding authors.
